# Pioneer to exploration and construction of Jilin Province’s higher medical laboratory education and talent training system

**DOI:** 10.1007/s13238-020-00719-5

**Published:** 2020-05-11

**Authors:** Huiming Han, Xichao Han, Sainan Wu, Baihui Lin, Xu Cheng, Qinlong Hou, Yongmei Li

**Affiliations:** 1 Medical College, Beihua University, Jilin, 132013 China; 2 The Center for Infection and immunity, Beihua University, Jilin, 132013 China; 3 The School of Life Sciences, Jilin University, Changchun, 130012 China

Professor Tingbin Yang (杨廷彬) has dedicated his whole life to education science and microbiology research. He is not only an excellent microbiologist but also an outstanding educator. He has finished many professional books and got several honors for his job. Prof. Yang has contributed a lot for the development of medical laboratory education in China and clinical medicine.

**Figure 1:**
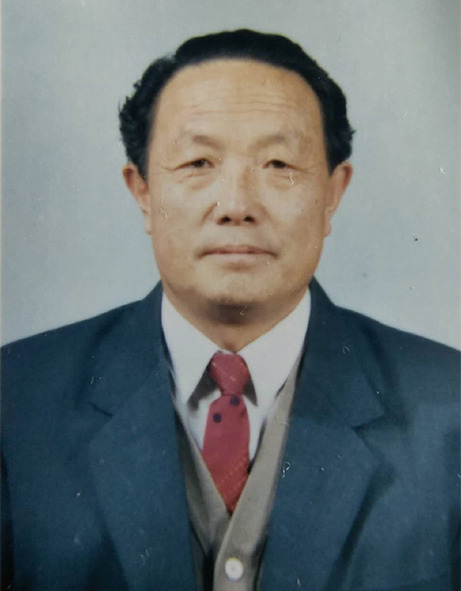
Professor Tingbin Yang

Prof. Yang was born in Yushu County, Jilin Provincein February, 1938 (Fig. 1). He suffered from the childhood days that are quite different from nowadays. However, Prof. Yang still didn’t give up on the pursuit of knowledge even in such situation. He believed firmly that war was the necessary way to get peace, but the developing science and technology was the best way to avoid wars. Supported by this belief, he concentrated on his school work and made progress in knowledge as time passed. Finally, in September, 1957, he was admitted to Dalian Medical College. During that time, he studied very hard and showed great diligence and eagerness. Finally, he graduated with impressive grades and accepted by Institute of Microbiology, Chinese Academy of Sciences as a graduate student. He finished his study in two years, and then worked in Microbiology Laboratory of Jilin Medical College in his hometown. In August, 1992, he worked in the Immunological Research Laboratory of Jilin Medical College, in October of the same year, he worked in the Immunological Laboratory of Dalian Medical College as the adjunct professor.

Prof. Yang promoted the countrywide education of higher medical laboratory technology in 1980. Medical laboratory technology is a science that examines materials from human body about microbiology, immunology, biochemistry, genetics, hematology, biophysics, cytology and so on, such as to provide information for prevention, diagnosis, treatment of human diseases and evaluation of human health. Supported by headmaster Zhi Liu (刘智), Prof. Yang visited Beijing several times and got the authority to be the first college which have the undergraduate courses on medical laboratory by the State Education Commission. In this way, more and more undergraduates had known how to identify human’s blood type, make a judgement on whether the patient is anemic, the liver’s function is normal or not and can even get clear about how to use several kinds of photoelectric instruments to finish the experimental analysis. Those were all thanks to the systematic learning during the undergraduate period. What’s more, students could also get the chances to go to some famous hospitals in Beijing for practice so that they could learn the newest and the most advanced medical technology and the management model. Finally, the students had broadened their horizons and improved knowledge learning. As a result, this was a great contribution to the reserve of national talents. The school has achieved a solid transformation from a specialized training class to a laboratory department, completed the undergraduate enrollment and master’s degree authorization, experienced a rapid growth in all aspects, trained a large number of laboratory medical workers working in the clinical and scientific research, and made important contributions to the development of the school and medical education. The reputation of Jilin Medical College had been promoted by Prof. Yang’s influence, in the meanwhile, many Medical Colleges in Hebei, Tianjin started to follow this education system.

While carrying out the educational reform, Prof. Yang also did excellent work on his scientific research and the professional book publishing. From 1983 to 1998, these books had been published successively, such as *Clinical microbiological test*, *Clinical Immunology*, *Dictionary of Medical Laboratory*, *Practical Immunology* and *Immunology and Immunoassay* (Yang Tingbin et al., 1982), of which Prof. Yang had participated in all of these books’ editorial works. It’s worth noting that he also published many papers at the same time. In 1985, Prof. Yang won the first prize of Jilin Science and Technology Association by the work *Research Report on Bacterial Change in the Treatment of Methylmercury-containing Wastewater by Biological Rotary Disk Method*. The theoretical basis and technical feasibility about treating the methylmercury wastewater by pseudomonas were discussed. He was not the only one who paid attention on the treatment of methylmercury wastewater, Shumin Yang (杨淑敏), Masuru Yamada and Tonomura Kenzo also had some ideas (Tonomura K et al., 1969. Yamada, M et al., 1971). Put all these ideas together, Prof. Yang decided to use the theory of Mercury-resistant strains’ repeated application in Methylmercury wastewater treating installation as the foundation. On this basis, He had proposed a simulation experiment of treating industrial wastewater containing methylmercury with *Pseudomonas* sp. P44 by biological turntable method (Yang Tingbin et al., 1981). With the continuous operation for nearly ten months, he proved that the bacteria can not only grow well in industrial wastewater containing methylmercury, but also keep its high efficiency of removing methylmercury, inorganic mercury and reducing COD. With the research of Prof. Yang, the methylmercury wastewater treatment had gotten a complete set of industrial processes distinguished from the previously published methods. In addition, his works were more remarkably effective, but were cheaper and more convenient.

Prof. Yang’s achievement was not only confined to environmental microbiology, but also related to clinical medicine. He used to publish several important research articles in this area. In 1992, he pointed out that the increase of the expression rate and intensity of HLA-DR^+^ monocytes were related to the aggravation of active SLE patients in his paper named *The Research of the Expression Rate and Intensity of HLA-DR Positive Monocytes in Peripheral Blood of SLE Patients* (Li Dao et al., 1992). In this study, the expression of HLA-R^+^ monocytes in peripheral blood of healthy persons and SLE patients was observed by alkaline phosphatase-antialkaline phosphatase (APAAP) bridging immunohistochemical method (Hu Jingxin et al., 1994). The chromaticity of HLA-R^+^ monocytes was measured by image chromaticity analyzer, and the expression intensity of HLA-R antigen was quantitatively analyzed. His Research had gotten the Achievement Award of Jilin Science and Technology Commission at that time. In the second year, his research about the immune mechanism of schizophrenia supported by National Natural Science Foundation got the Liaoning Science and Technology Progress Award. The paper holds that the *c-fos* gene of lymphocyte is associated with the immunological changes of schizophrenia. In order to prove his theory, he had listed a series of the experimental data, and he also made a discussion of the possibility of several other mechanisms of action at the end of the paper. This had provided a good foundation and a definite direction for the new coming research.

Prof. Yang was a rigorous and diligent scientist. He devoted himself to the science and education all his life, which proved a famous Chinese poem “Even the people with great ambition got old, but he could work harder to make the dream come true”. He still kept working with the challenges of science and education in his old age even though he had been seriously ill. He got the award for Scientific and Technological Achievements issued by the Ministry of Education of the People’s Republic of China by his *Experimental Diagnostic Techniques of Schizophrenia Based on Serum Analysis*. We may get elder, but our ideal should not be. Prof. Yang’s story has inspired me all the time. We should respect such an outstanding scientist like him forever.
